# Dissection of the Regulatory Elements of the Complex Expression Pattern of Puckered, a Dual-Specificity JNK Phosphatase

**DOI:** 10.3390/ijms222212205

**Published:** 2021-11-11

**Authors:** Katerina Karkali, Enrique Martin-Blanco

**Affiliations:** Instituto de Biología Molecular de Barcelona (IBMB-CSIC), Parc Cientific de Barcelona, Baldiri Reixac 10-12, 08028 Barcelona, Spain; kkabmc@ibmb.csic.es

**Keywords:** JNK, *Drosophila*, dual specificity phosphatase, gene expression

## Abstract

For developmental processes, we know most of the gene networks controlling specific cell responses. We still have to determine how these networks cooperate and how signals become integrated. The JNK pathway is one of the key elements modulating cellular responses during development. Yet, we still know little about how the core components of the pathway interact with additional regulators or how this network modulates cellular responses in the whole organism in homeostasis or during tissue morphogenesis. We have performed a promoter analysis, searching for potential regulatory sequences of *puckered (puc)* and identified different specific enhancers directing gene expression in different tissues and at different developmental times. Remarkably, some of these domains respond to the JNK activity, but not all. Altogether, these analyses show that *puc* expression regulation is very complex and that JNK activities participate in non-previously known processes during the development of *Drosophila*.

## 1. Introduction

Developmental biology focuses on the analysis of the events occurring during the development of the organism. It aims to understand the mechanisms directing how, from a single cell, multicellular organisms, with their extraordinary variety of shapes and forms, are built through complex morphogenetic processes.

Signaling pathways are key for morphogenesis as they transduce extracellular information regulating cell proliferation, determining developmental axis, specifying compartment domains, or modulating cell shape or apoptosis. Remarkably, the number of these pathways is finite. A few signaling modules are activated once and again by divergent stimuli to elicit context-specific cellular responses. To understand how the same transducing cascades regulate so many different events is fundamental to gain insight into how both their activity and their target genes are controlled. The recent literature points to new emerging ideas, considering these pathways more a subject of threshold events and regulatory feedback loops than of on/off signaling switches [1]. It has, thus, become crucial to understand when, where, and how these pathways are activated, for how long this activation lasts, which is their level of activity and how is this dynamically controlled in time and space.

JNK is an acronym for the c-Jun-N terminal kinase. The JNK belongs to the family of MAPKs (Mitogen Activated Protein Kinases) like ERK (Extracellular Signal Regulated Kinase) and p38 Kinase [2]. Originally, the JNK signaling pathway was identified as a stress-response kinase cascade controlling cell survival and proliferation [3]. It is highly conserved throughout evolution and homologs for JNK have been identified in mice, humans, zebrafish, *C. elegans* and *Drosophila* [4]. The JNK pathway is essential for the amplification of cellular responses to growth factors and cytokines, controls cell shape, proliferation, adhesion and death; and regulates a plethora of different fundamental processes like stress, innate immune response and apoptosis [5]. During development, signaling mechanisms regulated by JNK have been shown to modulate epithelial fusion [6] and the cohesion of border cell clusters during migration [7] in *Drosophila*, as well as multiple morphogenetic processes and wound healing in vertebrates [8,9]. It has also a role in many different pathological conditions, such as atherosclerosis, Parkinson’s or Alzheimer’s disease [10,11].

The JNK signaling responds to many different stimuli of physical and chemical nature, e.g., heat, mechanical, oxidative and osmotic stress [12,13]; growth factors [14,15], or insulin [16]. However, the information on the stimuli and receptors involved in activating JNK is far from being saturated, since they vary largely depending on the physiological context and the cell type. It would be extremely important to define the interaction between the core components of the pathway and additional regulators to understand how this genetic network adequately modulates cellular responses in culture but also in the whole organism in homeostasis or during tissue morphogenesis.

In *Drosophila* the core of the cascade consists of the homologs of JNKK and JNK, which are encoded by the genes *hemipterous* (*hep*) [17] and *basket* (*bsk*) [18]. Mutant analysis has revealed that the entire JNK cascade participates in the completion of dorsal closure (DC) in embryonic stages. Mutants for *hep*, *bsk* or *kayak* (*kay*) (Fos in *Drosophila*) display embryonic dorsal holes [19]. When JNK signaling is defective, the leading-edge (LE) cells of the embryonic lateral epidermis slightly elongate but quickly revert to polygonal shapes. They fail to accumulate actin and myosin at the dorsal most edges and as a consequence, they do not extend filopodia and the actin cable, inherently built at the edge of the epithelia, is not assembled [20]. A restricted activation of the JNK cascade is required for the differentiation of the leading-edge cells during DC, where the JNK signaling pathway controls the expression of the gene *puckered (puc)*; *puc* is an immediate-early gene responding to JNK activity, that encodes a dual-specificity MAPK phosphatase that acts to down-regulate DJNK/Bsk activity through dephosphorylation [21]. The expression of Puc in the leading-edge cells results in the establishment of a negative regulatory feedback loop [22]. In *puc* mutants, the activity of JNK becomes upregulated, LE cells accumulate higher levels of actin and myosin, and they undergo excessive progression resulting in puckering of the epidermis at the dorsal midline [20].

We previously performed a promoter analysis searching for potential regulatory sequences of *puc* and identified an enhancer specific for border cells expression during *Drosophila* oogenesis [7]. Here we present a general overview of the results of this screening. Besides identifying the role of the JNK pathway in the collective-cell movements of border cells, we isolated multiple regulatory domains in *puc* directing gene expression in different tissues and at different developmental times. Remarkably, some of these domains respond to the JNK activity, but not all. Altogether, these analyses point to a complex scenario in which *puc* and JNK activities seem to be required in many non-previously anticipated events during the development of *Drosophila*.

## 2. Results

*puc* low-level expressivity, as well as the high instability of its transcripts, have prevented direct observation of its expression; with the exception of in situ detection at the epidermal leading edge in embryos during dorsal closure and in follicle cells [21,23,24,25,26]. No antibody is available and the expression of *puc* in other tissues has been inferred by studying different enhancer trap P elements inserted in or around the gene. *puc* expression has been reported in: (1) a population of lateral cells (LE cells) that delineates the boundary between the ectoderm and the amnioserosa during dorsal closure [21]; (2) the amnioserosa itself (transiently) [27]; (3) the peripheral embryonic nervous system [21,28]; (4) discrete neurons and neuronal precursors in the embryonic central nervous system (CNS) [26]; (5) epidermis and spiracles in the third instar larva [21]; (6) specific cell populations in all thoracic imaginal discs (the proximal part of the wing, haltere and leg discs, in the stalk, where imaginal discs connect to the larval epidermis and in the peripodial epithelium) [6,24]. Later during prepupariation, *puc* expression is maintained in the peripodial membrane and marks the presumptive suture sites of imaginal discs with their neighbors [29]; (7) all photoreceptor precursors (very weak) [30,31]; (8) sensory organs precursors in the wing, leg and haltere imaginal discs (weak) [32]; (9) larval muscles (very strong expression) [22]; (10) different types of follicular cells and the border cells in egg chambers [7,25]; (11) embryonic and larval hemocytes [33]; (12) induced at the edge of wounds in epithelial cells in embryos, larval epidermis and imaginal discs [34,35]; (13) epithelial regions surrounding necrotic areas [36]; (14) larval midgut [37]; and the adult brain [38].

In most locations, the expression of *puc* depends on the activity of the JNK signaling cascade and upstream regulators (reported in most of the references above). However, in discrete events, *puc* expression has also been observed in the absence of JNK activity, in particular in the larval epidermis and posterior spiracles [21] and in necrotic cells in dorsal open embryos in *hep* and *bsk* mutant conditions (E. Martin-Blanco, unpublished results). ln order to identify the regulatory elements involved in controlling the expression of *puc*, we developed different approaches. We analyzed the ability of different genomic fragments (PGs) in and around the gene to drive the expression of a reporter. Further, we performed an evolutionary analysis in silico and identified highly conserved regions that we also tested as potential regulatory sequences (RSs).

### 2.1. puc Genomic Domain Analysis

**Figure 1:**
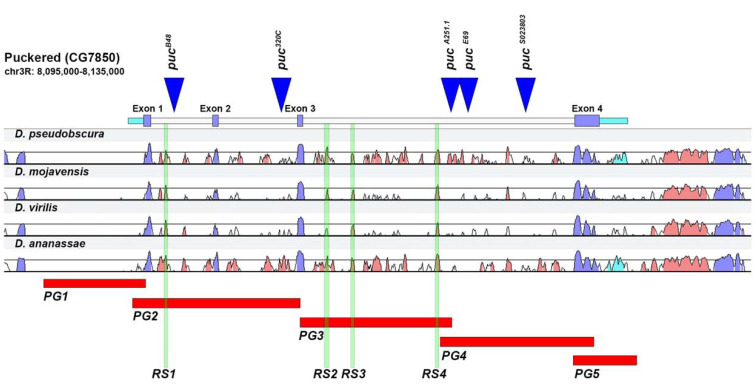
*puc* genomic organization. The area expanding the gene *puc* expands around 26 Kb. It includes four exons (purple) that are conserved across all *Drosophilae*. Several P element insertions have been identified within the gene; those with known expression patterns (enhancer traps) or phenotypically characterized, have been mapped (blue triangles). For characterizing the *puc* genomic region, we have subdivided it into five domains [red boxes—*puc* Genomic Regions 1 to 5 (PG1 to PG5)]. These domains covered: upstream sequences and the 5′ untranslated domain (PG1); the first and second intron (PG2); the 5′ half of the third intron (PG3); the 3′ half of the third intron (PG4); and the 3′ untranslated domain and downstream sequences (PG5). We further identified four hyper-conserved motifs (Regulatory Sequences—green vertical bars) in *puc* introns shared in *D. mojavensis*, *D. virilis* and *D. ananassae*, the most divergent species from *D. melanogaster* sequenced so far. RS1 resides within the PG2 domain and RS2–4 map in the PG3 region.

*puc* cytologically maps on the right arm of the third chromosome (84E12–84E13) and its transcription unit comprises four exons. The P element insertions isolated so far are all inserted in different introns and exhibit different patterns of expression (Figure 1). This suggests that the intronic regions of *puc* can contain distinct enhancers regulating differential subsets of *puc* expression. The different P element insertions in homozygosis also show different phenotypes. Thus, the B48 allele inserted in the first intron reproduces a different pattern of expression in follicle cells and in embryos during dorsal closure, than the A251 insertion, which is localized in the third intron [21].

We have subdivided the *puc* genomic region into five domains [*puc* Genomic Regions 1 to 5 (PG1 to PG5)]. These domains covered: upstream sequences and the 5′ untranslated domain (PG1); the first and second intron (PG2); the 5′ half of the third intron (PG3); the 3′ half of the third intron (PG4); and the 3′ untranslated domain and downstream sequences (PG5). The candidate regulatory domains (PG1 to PG5) were cloned in the PPTGal4 vector and transgenic lines were created to study their regulatory effects in vivo (Figure 1).

To extend and refine our analysis of *puc* regulatory domains, we decided to make use of available bioinformatic tools to search for possible regulatory regions. The bioinformatic search was restricted to the intronic regions of *puc*. First, a comparative analysis to identify conserved regions was done using the Vista point tool (see Methods). The comparison was performed with three species chosen based on their phylogenetic distance to *D. melanogaster (D. pseudobscura, D. virilis* and *D. mojavensis)* (Figure 1).

The four most conserved regions within the introns (RS1 in the domain covered by PG2, and RS2 to RS4 within the PG3) were scanned with the Position-Weight Matrices (PWM) of the TRANSFAC database in order to associate them to known transcription factors (Match Suite) (see Methods). RS2 was the only conserved region that got a statistically accepted score. The matrices that theoretically bind this region are those involving c-Jun, Creb and CRE-BP1/c-Jun heterodimer. The rest of the conserved regions did not get any matrix associated. We also performed a less stringent scan analysis (at 85% relative profile score threshold) employing JASPAR *D. melanogaster* non-redundant transcription factor (TF) binding profiles database (see Methods). This survey provided information on potential binding motifs for the 4 RS domains, beyond confirming the presence of c-Jun and Creb sites in RS2 (Table S1). A number of sites, with potential regulatory relevance, for different transcription factors of distinct subfamilies were identified (see Discussion). These sequences may keep important functional roles considering their high level of conservation. The candidate regulatory sequences (RS1 to RS4) were cloned as for the PG lines in the PPTGal4 vector and transgenic lines were built (Figure 1).

### 2.2. Analysis of Lines Carrying puc Regions In Vivo

To test the different Gal4 transgenic lines generated (PG1–PG5 and RS1–RS4), they were crossed with a multimerized UAS-GFP stock [39]. Following the GFP expression during fly development allowed us to determine if these lines reproduced the expression pattern of *puc*, partially or completely. For each of the available Gal4 lines, expression was evaluated in embryonic tissues, in first instar larval brains and in third instar full larvae.

### 2.3. Upstream Sequences (PG1)

**Figure 2:**
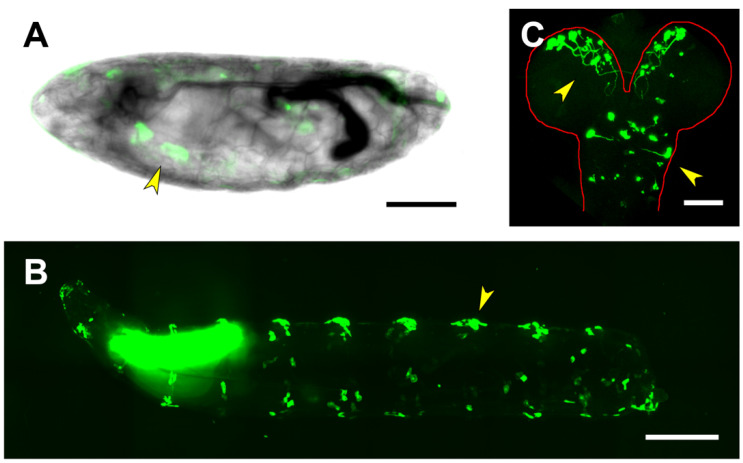
Expression directed by the Upstream Regulatory Sequences of *puc*. (**A**) Expression of a PG1-directed GFP-reporter on a live, late stage 17 embryo. Salivary glands (arrowhead) and conspicuous groups of epithelial cells are labeled. Scale bar 100 µm. (**B**) Lateral view of a live third-instar larva, showing strong expression of the PG1-directed GFP-reporter in the salivary glands and in segmentally iterated groups of epithelial cells at the locations of the dorsal denticles (arrowhead). Expression is also observed in epithelial cells distributed in scattered ventral spots. Anterior is left. Scale bar 400 µm. (**C**) Representative image of GFP-immunostained first-instar larva brains, dissected from animals expressing the GFP-reporter under the control of the PG1 Gal4 line. Arrowheads point to PG1-driven expression in neurons of the brain lobes and the thoracic ganglion. The CNS perimeter is marked (red line) and the anterior is up. Scale bar 20 µm.

The 4kb region comprising the *puc* transcription start site plus its 5’ untranslated domain is not carrying relevant transcriptional regulatory elements for embryonic development. None of the different insertions of the PG1 construct show detectable expression, neither in embryonic tissues nor in first instar larvae (beyond a faint signal in the salivary glands and in the posterior spiracles) (Figure 2A). Yet, GFP expression could be detected in neurons of the brain lobes and the thoracic ganglion in dissected and immunostained first instar larvae CNS (Figure 2B). This expression does not sustain at later stages and no signal is detected in third instar larval brains. Third instar larvae show strong expression on the salivary glands and on iterated group of cells corresponding to those involved in secreting the larval cuticle denticles (Figure 2C).

### 2.4. First and Second Intron (PG2, RS1)

**Figure 3:**
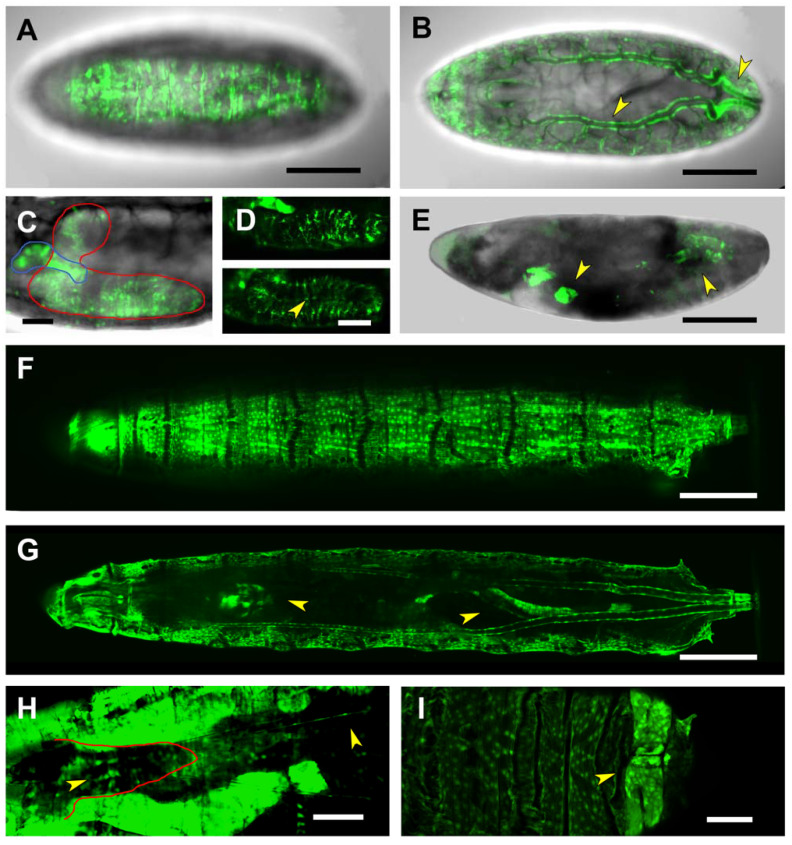
PG2-driven expression in embryonic and larval stages. (**A**) and (**B**). Lateral (**A**) and dorsal (**B**) views of a live, late-stage 17 embryo, expressing GFP under the control of the PG2 Gal4 line. Expression is detected throughout the epidermis (**A**), in the tracheal dorsal trunk wall, as well as in the posterior spiracles (arrowheads in (**B**)). Scale bar 100 µm. (**C**) Image corresponding to a deeper focal plane of the embryo shown in (**A**). GFP-expression is observed in the foregut in between the two brain lobes (demarked by a blue line) as well as in the CNS (demarked by a red line). Scale bar 20 µm. (**D**) Ventrolateral (upper panel) and mediolateral (lower panel) views of the ventral nerve cord portion of the CNS are shown in (**C**). Activation of the PG2 fragment in the CNS is limited to the perineural/sub-perineural glia and the channel glia (arrowhead). Scale bar 20 µm. (**E**) Lateral view of a stage 17, maternal-zygotic *hep* mutant embryo, with a characteristic dorsal open phenotype. Reporter activation by the PG2 fragment remains unaffected in the remnants of the salivary glands, the dorsal tracheal trunk and the posterior spiracles (arrowheads). No expression was observed in the epidermis. Scale bar 100 µm. (**F**) and (**G**). Lateral (**F**) and dorsal (**G**) views of a live third instar larva, exhibiting strong GFP expression in the epidermis and the dorsal tracheal trunk (**F**), as well as in the brain and in the midgut (arrowheads in (**G**)). In the nervous system, puc expression is limited to two rows of cells running along the VNC and to a limited number of glia ensheathing intersegmental nerves (**H**). Last, it is expressed in the anal pad (**I**). Scale bar 400 µm. In all cases, anterior is left.

The first and second introns of *puc*, as well as its second exon, are represented by the PG2 Gal4 line. This region appears to enclose different regulatory elements active in different tissues and periods. PG2-driven expression was first detected in the CNS of stage 15 embryos and patchy at the whole epidermis (Figure 3A). As development proceeds, expression was also observed all over on the tracheal system and at the posterior spiracles (Figure 3B). PG2 was additionally found to be active in a group of cells positioned in between the brain lobes, that could correspond to the foregut (Figure 3C,D).

**Figure 4:**
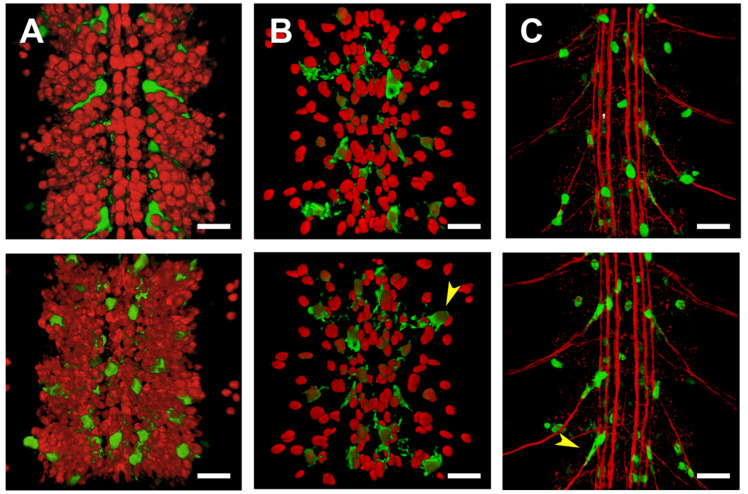
PG2 is expressed in glia. (**A**–**C**) Flat preparation of embryos, at stage 17, expressing GFP under the control of the PG2 Gal4 line. Dorsal (top) and ventral (bottom) views of three segments of the ventral nerve cord (VNC) are shown. (**A**) Double staining with an anti-ELAV pan-neural antibody. (**B**) Double staining with an anti-Repo antibody labeling glial cells. Arrowhead points to cells co-expressing GFP and Repo (Glia). (**C**) Double staining with an anti-Fasciclin 2 antibody marking the longitudinal connectives, segmental and intersegmental nerves. Arrowhead points to an ensheathing glial cell expressing PG2-directed GFP. Scale bar 10 µm. In all cases anterior is top.

The activity of the PG2 regulatory elements in the CNS was further analyzed by double immunostaining experiments performed in flat-dissected, stage 16 embryos. The GFP reporter co-localized with Repo, a transcription factor involved in glial differentiation, but not with the pan-neuronal marker Elav, indicating that PG2 was active in the embryonic CNS glia (Figure 4A,B). These glial cells were, by positional criteria, identified as perineural/sub-perineural [belonging to the subgroup that forms the Blood-Brain Barrier (BBB)] and ensheathing glia (Figure 4C). The CNS expression observed in the late embryonic stages persisted in the first instar larvae. The number of glial cells activating the PG2 sequence increases considerably (Figure S1).

In the third instar larvae, the expression directed by the PG2 domain is sustained for most tissues, the epidermis, the tracheal system, the posterior spiracles and the foregut (Figure 3E,F). In the nervous system, it is refined to two rows of cells running along the VNC and to a limited number of glia ensheathing the intersegmental nerves (Figure 3H). Further, it becomes prominent in the posterior midgut (Figure 3G) and the anal pad (Figure 3I).

In summary, the first and second introns of *puc* appear to be active regulatory domains probably containing multiple regulatory elements. In a first approach to identify these elements we took advantage of our genomic comparative analysis and explored the expression patterns generated in response to the RS1 motif, which as described above is one of the four hyper-conserved genomic regions found in *puc*. RS1 maps in its first intron within the PG2 regulatory domain (Figure 1). Expression driven by this line is detected in late embryonic stages, in the salivary glands; in some epidermal cells, in the posterior midgut/hindgut and in the posterior spiracles. It is not activated in the embryonic CNS. RS1 is thus reproducing, in a very limited way, the activation pattern of the PG2. Remarkably, it shows expression in a group of cells not found for PG2 around the maxillary primordia. (Figure S2A). In the third instar larva, RS1 also fails to recreate the expression observed for PG2. Its activity is restricted to scattered cells in the epidermis, a partial expression in the tracheal system and to the posterior spiracles. The premaxillary group of cells expressing RS1 in embryos are still detected at this stage (Figure S2C,D).

### 2.5. Third Intron (PG3, PG4, RS2–4)

The examination of *puc*’s long third intron for residing cryptic enhancers was facilitated by splitting its sequence into two fragments and by the generation of the lines PG3 and PG4, with PG3 corresponding to the 5′ portion of the intron. The PG3 domain covers the three hyper-conserved motifs RS2, RS3 and RS4 (Figure 1).

**Figure 5:**
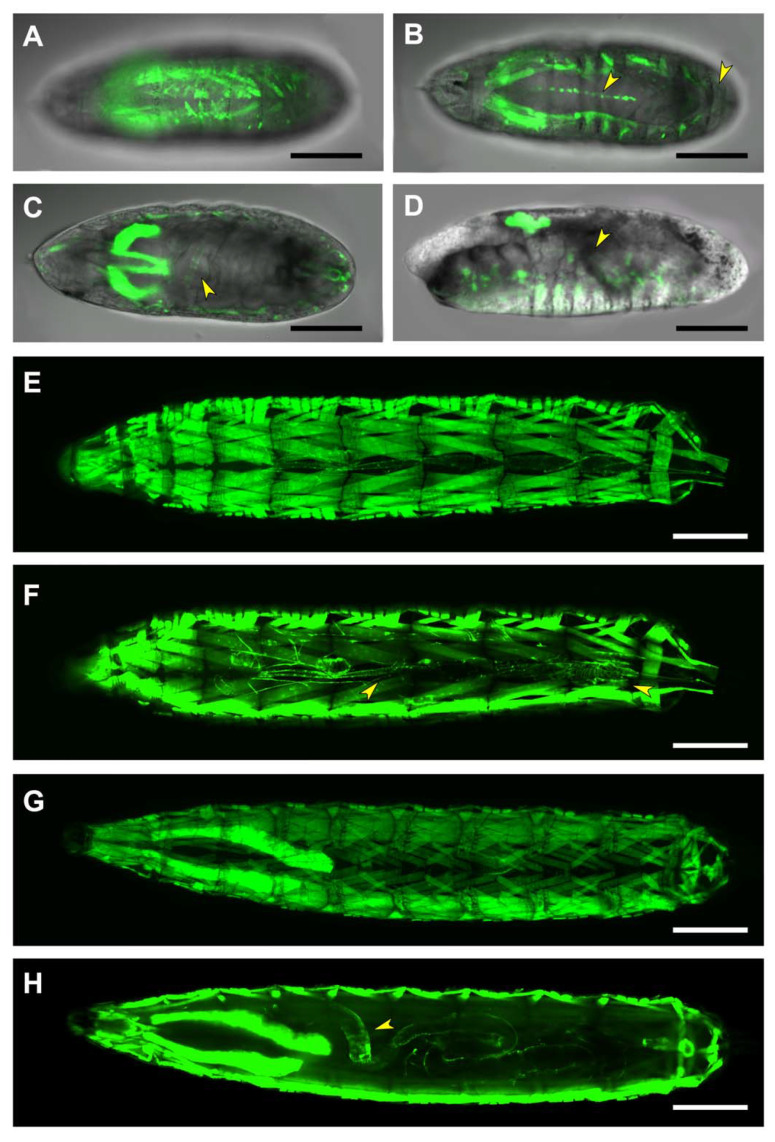
Gene expression modulation by the PG3 domain. (**A**–**C**) Ventral views, acquired at different focal depths (with dorsal directionality), of a live, stage 17 embryo, expressing GFP under the control of the PG3 Gal4 line. Expression is detected in all body wall muscles (**A**); in the CNS midline glia (arrowhead in (**B**)); and in the salivary glands, pharynx, intestinal tract and anal pad (arrowheads in (**C**)). Scale bar 100 µm. (**D**) Lateral view of a dorsal-open, stage 17, *hep* null embryo. The activation of the PG3 regulatory sequence, in the salivary glands and in the remnants of the muscles (arrowheads) is sustained despite the loss-of-function of the JNK-activating kinase Hep. Scale bar 100 µm. (**E**) and (**F**). Dorsal superficial and deep views of a live, third instar larva. PG3 exhibits strong activation in all body-wall muscles (**E**). In a deep focal plane (**F**), PG3 activity is revealed in the heart tube, as well as in the alary muscles (arrowheads in (**F**)). Scale bar 400 µm. (**G**) and (**H**). Ventral superficial and deep views of a living third instar larva. GFP-expression directed by PG3 is detected in the salivary glands (**G**) and in the intestinal tract (arrowhead in (**H**)). Scale bar 400 µm. In all cases, anterior is left.

Expression driven by the PG3 line is detectable only from late embryonic stages (stages 16–17), in the salivary glands, the pharynx/ring gland and weakly in the midgut and the anal pads. Yet, its most characteristic feature is to be expressed in all body wall muscles and the nervous system (Figure 5A–C). Its expression in the embryonic CNS is distinct from that found for PG2. It is restricted to the Repo-negative midline glia (Figure S3A,B) shifting in the first instar larval brain to the surface glia. Here, the expression in surface glia becomes weak when compared to a massive expression observed in the tracheal branches penetrating the brain (Figure S3C,D).

In the third instar larvae, the expression directed by the PG3 domain in muscles recapitulates that observed in the embryo (Figure 5E). Further, the heart and alary muscles (other mesodermal derivatives) are also stained (Figure 5F). The expression in the nervous system disappears while it gets strengthened in the midgut extending to the hindgut, and the salivary glands (Figure 5G,H).

**Figure 6:**
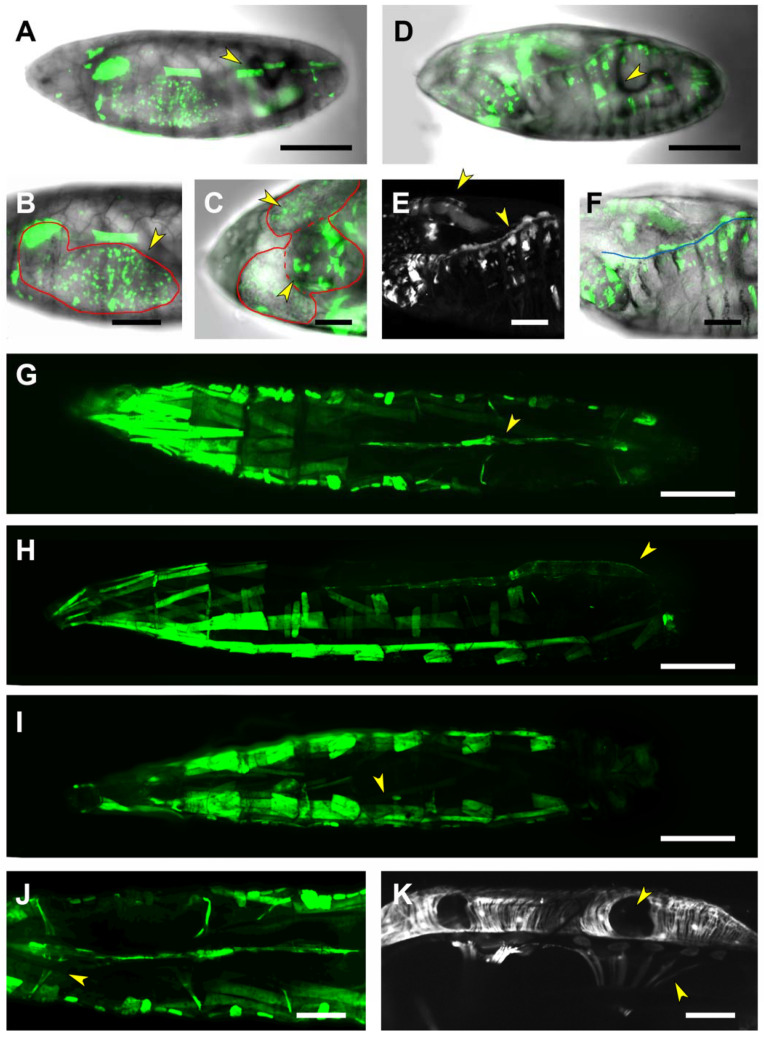
Complex gene expression modulation by the RS2 motif. (**A**) Lateral view of a live, stage 17 embryo expressing GFP under the control of the RS2 motif. Expression is detected in a subset of body wall muscles (arrowhead), the CNS and, faintly, in the posterior midgut. Scale bar 100 µm. (**B**) and (**C**) high magnification views (**B**) lateral and (**C**) ventral of the embryonic CNS (delimited by a red outline) of (**A**). Arrowheads point to neurons in the optic lobes and perineural and subperineral glia on the VNC. Scale bar 20 µm. (**D**) Lateral view of a dorsal-open, stage 17, *hep* null embryo. The activation by the RS2 motif in the remnants of the muscles, the CNS and the midgut is sustained (arrowheads). Scale bar 20 µm. (**E**) and (**F**) high magnification views showing the GFP signal (**E**) or the signal in a brightfield background (**F**) of the epithelial surface of (**D**). Arrowheads point to the ectopic expression of the marker in the anterior midgut and the epidermal cells (blue line) at the edge of the open hole consequence of the dorsal closure failure in hep mutants. Scale bar 20 µm. (**G**–**I**) Dorsal (**G**), lateral (**H**) and ventral (**I**) views of a live third instar larva showing RS2-directed GFP expression in a subset of body-wall muscles, in the heart tube and in the alary muscles (arrowheads). Scale bar 400 µm. (**J**) High magnification image of the heart tube and the attached alary muscles (arrowhead) expressing RS2-directed GFP. Scale bar 100 µm. (**K**) 3D-reconstruction of the heart tube and the attached alary muscles (asterisks). Arrowheads point to the hemolymph exit pores along the cardiac tube. Scale bar 100 µm. In all instances, anterior is left.

Three out of the four identified hyper-conserved *puc* sequences (RS2–4) map in the PG3 intronic fragment. The complex embryonic expression pattern observed for the PG3 domain is only in part reproduced by the RS2 to RS4 conserved sequences. The RS2 motif, residing at the 5′ end of the PG3 sequence, is activated at the end of embryogenesis (late stage 17) in the salivary glands and in a subset of the lateral body wall muscles, in the posterior spiracles and in the CNS (Figure 6A–C). In the CNS, RS2-directed expression is found in both neurons and glia (Figure S4A–D) persisting in the first instar larva stage glia (Figure S4E,F). In the third instar larva, the RS2-directed GFP expression persists in a fraction of body-wall muscles, in the heart tube, in the alary muscles and at the periphery of the anal pad (Figure 6E–G). No expression is detected in the nervous system. The expression of RS2 in the heart and auxiliary structures are restricted to mesodermal derivatives all along the anterior-posterior axis of the heart tube (Figure 6H,I).

**Figure 7:**
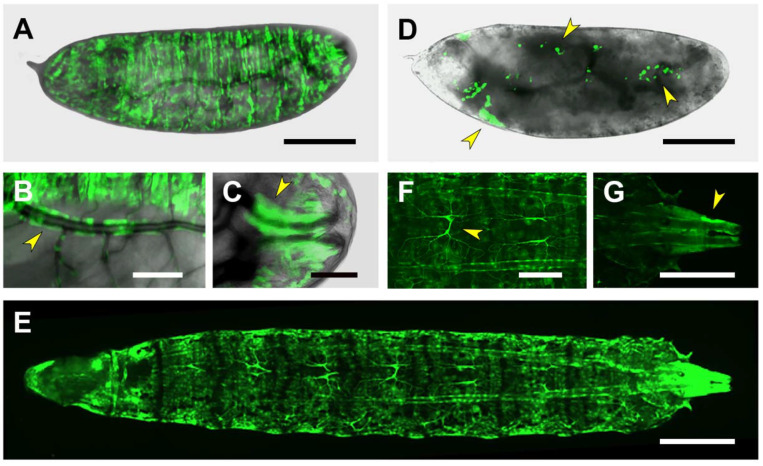
RS3-driven expression in embryonic and larval stages. (**A**) Dorsal view of a live, stage 17 embryo, expressing GFP under the control of the RS3 Gal4 line. Strong activation of this motif is observed throughout the epidermis. Scale bar 100 µm. (**B**) and (**C**) Details from the embryo shown in (**A**). RS3 activation is observed in the trachea wall (arrowhead in (**B**)) and in the posterior spiracles (arrowhead in (**C**)). Scale bar 20 µm. (**D**) Lateral view of a dorsal-open, stage 17, *hep* null embryo. In *hep* null embryos, activation of the RS3 motif is abolished from the epidermis but not from the trachea. Arrowheads point to remnants of the trachea that sustain RS3-induced GFP expression. Scale bar 100 µm. (**E**) Dorsal view of a live third-instar larva, showing strong expression of the RS3-directed GFP-reporter in the epidermis, the trachea and the posterior spiracles. Scale bar 400 µm. (**F**) and (**G**) High magnification images showing details of RS3 expression in third instar larval tracheal system. Arrows point to the tracheal terminal arborizations at the dorsal midline (**F**) and at the posterior spiracles (**G**). Scale bars 20 (**F**) and 50 µm (**G**) respectively. Anterior is left.

RS3 directed expression is quite distinct from that resulting from the PG3 domain. RS3, in embryos at late-stage 17, besides the salivary glands, targets expression to the posterior spiracles, the trachea and to the epidermal cells (Figure 7A–C). No CNS or mesodermal expression was observed. In third instar larva, the RS3-directed GFP expression in the epidermis and the posterior spiracles is maintained. The tracheal expression becomes more complex, and it is not limited to the main trunks. The most distal tracheal cells do now display, at high levels, RS3-directed expression (Figure 7E–G).

RS4 expression in embryonic stages is limited to the salivary glands and the posterior spiracles, not showing any of the characteristic patterns observed for PG3, except a very faint expression at the ventral midline in the VNC (Figure S5A,B). In third instar larva, RS4 becomes active at the maxillary region, the tracheal trunks, scattered epidermal cells, the anal pad, and weakly at the posterior midgut and the CNS (Figure S5C,D).

**Figure 8:**
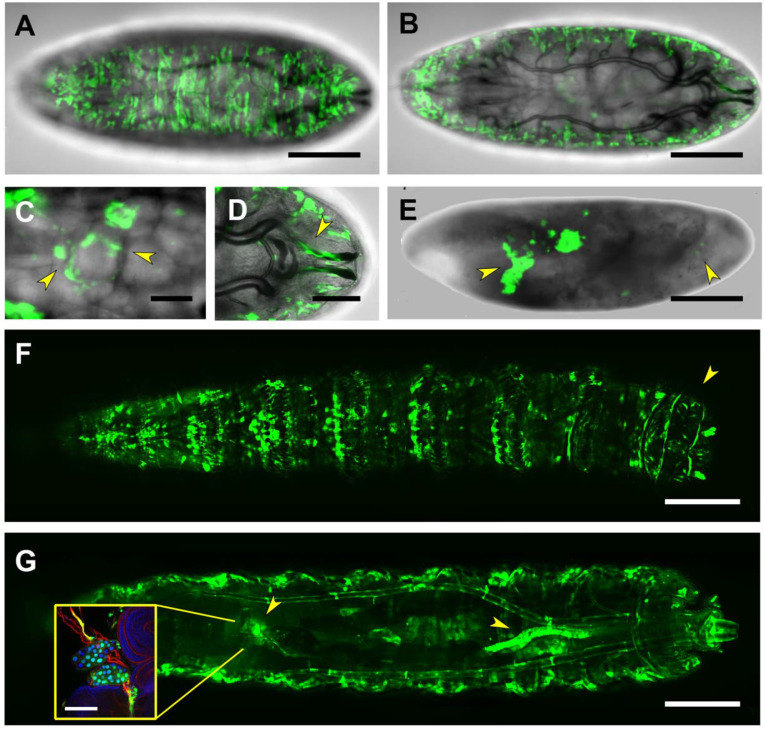
Gene expression modulation by the PG4 domain. (**A**) and (**B**) Dorsal views at different focal planes of a live stage 17 embryo showing strong epidermal GFP expression directed by the PG4 Gal4 line. Expression by this line is also driven in a group of 4–5 cells positioned medially along the embryo’s dorso-ventral axis (arrowhead in (**B**)). Scale bar 100 µm. (**C**) and (**D**). High magnification images from (**A**). PG4 is activated in the ring gland and anterior midgut (arrowheads in (**C**)); as well as in the posterior spiracles (arrowhead in (**D**)). Scale bar 20 µm. (**E**) Lateral view of a dorsal-open stage 17 *hep* null embryo, showing PG4 activation in the salivary glands and on a medially positioned group of cells and the remnants of the posterior spiracle (arrowheads). Scale bar 100 µm. (**F**) Ventral view of a live, third instar larva, exhibiting activation by PG4 sequence in the anal pad (arrowhead) and the epidermis. Scale bar 400 µm. (**G**) Dorsal view of a third instar larva showing distinct PG4-directed expression predominantly in the midgut and the ring gland (arrowheads) but also in scattered positions along the dorsal trachea trunk and posterior spiracles. Scale bar 400 µm. Inset shows a high magnification of the ring gland expressing GFP as a marker for PG4 activity. DAPI is blue and Phalloidin is red. Scale bar 20 µm. Anterior is left, in all instances.

The complex pattern targeted by the lines mapping at the 5’ half of *puc*’s third intron was not as such for the PG4 line, which covers the 3’ half of this intronic sequence. In the embryo, PG4 is activated at stage 15 and drives expression in the salivary glands and the epidermis (in a patchy pattern) (Figure 8A,B). Further, strong expression was also detected in a group of four to five cells, possibly components of the ring gland medially positioned along the embryo’s dorso-ventral axis in between the brain lobes, and in the posterior spiracles (Figure 8C,D). No expression was observed in the embryonic CNS. However, in the first instar larva, the PG4 sequence is activated in specific, interneurons of both the brain lobes and the VNC, as well as in a group of posterior neurons with axonal projections outside the CNS. A subset of astrocytes and ensheathing glial cells also appear to express the reporter (Figure S6A–C). In third instar larvae, no obvious expression was held in the CNS, but it was maintained in the epidermis and the posterior spiracles. The ring gland is heavily stained as they are the posterior midgut and specific cells at the trachea segmental junctions (Figure 8F,G).

### 2.6. Downstream Sequences (PG5)

**Figure 9:**
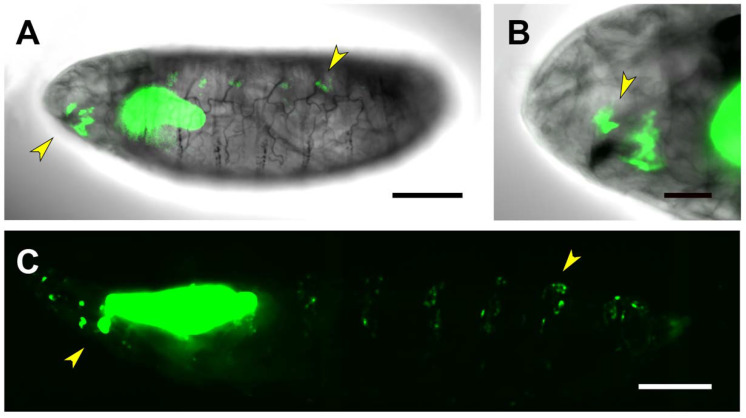
Gene expression modulation by the PG5 domain. (**A**) Lateral view of a live stage 17 embryo expressing GFP under the control of the PG5 Gal4 line. Expression is detected in the salivary glands, in cells surrounding the maxilla and in iterated groups of epidermal cells (arrowheads). Scale bar 100 µm. (**B**) High magnification of the image in A focus on those PG5 positive cells around the embryonic maxilla. Scale bar 20 µm. (**C**) Lateral view of a live third instar larva showing the persistence of the embryonic PG5 activation pattern. Arrowheads point to the cells surrounding the maxilla and the denticle bearing cells. Scale bar 400 µm. Anterior is left.

*puc* 3’ non-coding sequences examined (PG5 Gal4 line) includes its last exon, 3′UTR and sequences downstream. The PG5 sequence is activated during the last embryonic stage (stage 17) in the salivary glands and in the embryonic maxilla at the anterior tip of the embryo. Expression is also detected in segmentally iterated groups of cells, positioned dorsally and slightly posteriorly with respect to the brain lobes occupying symmetrical positions at the branching points of the tracheal dorsal trunk (Figure 9A,B). No expression was detected in the CNS. In third instar larvae, PG5 is active in the salivary glands, in a restricted group of cells positioned below the dorsal denticles and in the posterior spiracle (Figure 9C).

### 2.7. Regulatory Control of puc Expression by the JNK Signaling Cascade

As mentioned above, *puc* expression (detected from enhancer trap lines) has been thoroughly employed as a readout of JNK signaling activity. Yet, *puc* has, in places, been found to be expressed in the absence of JNK. We tested a subset of the new enhancers generated in our screening for dependence on JNK activity and evaluated the expression of these new lines in a *hep* minus maternal and zygotic background. A stock carrying the *hep^1^* mutation and a multimerized UAS-GFP inducible marker was built. Homozygous, maternally rescued, *hep^1^* females, of the aforementioned stock, were crossed to males of the Gal4 lines PG2 to PG4 and RS1 to RS3. Hemizygous *hep^1^* male embryos not maternally rescued were identified in the progeny by their characteristic dorsal open phenotype. GFP expression was monitored in these living embryos. Remarkably, much of the expression directed by the new enhancers is not JNK dependent.

For PG2, covering the first and second intron of *puc*, the *hep^1^* embryos display GFP expression in the remnants of the salivary glands and the trunks of the tracheal system. The epidermal expression seems to be reduced but some signal still remains (Figure 3E). No conclusion could be reached regarding the nervous system. The RS1 motif included in the PG2 domain, however, responds to JNK activity but in an unexpected way. In the absence of JNK activity, RS1-directed expression shows up strongly on the epidermis, while in the wild type condition is limited to scattered epidermal and tracheal cells and to a posterior domain associated with the spiracles.

The expression patterns on salivary glands, body wall muscles and the midgut directed by the 5′ fraction of *puc* third intron (PG3) are unaffected in *hep^1^* embryos (Figure 5D). It was very difficult to assess any difference of the PG3-directed nervous system or heart expression as these were, originally, quite low.

The patterns of expression of RS2 and RS3 enclosed within the PG2 domain respond differentially to JNK activity (we did not evaluate RS4 as its expression pattern is quite unspecific and remarkably low).

RS2-directed expression recapitulates in part the PG3 muscle pattern but not its tracheal expression. It also shows a quite distinct expression in the CNS, mostly in glial cells. In the absence of JNK activity, the muscle and CNS expression (as it happens for PG3) are maintained (Figure 6D). Interestingly, RS2 becomes ectopically expressed in the midgut and in a row of cells at the edge of the opening dorsal hole (Figure 6E,F). These last cells have been previously observed to activate *puc^A251.1^* and *puc^E69^* enhancer trap expression both in *hep^1^* and *bsk^2^* allelic conditions (unpublished observations and J. Riesgo-Escovar, personal communication).

The RS3-directed expression pattern is quite dissimilar to the PG3 one showing widespread epidermal and tracheal expression. Especially, the tracheal cells of the terminal branches and the posterior spiracles. In *hep^1^* mutants, remnants of GFP expression in the salivary glands and scattered cells that seem to be of tracheal origin are found. The epidermal and terminal tracheal cells expression seems to be abolished in the loss of JNK activity (Figure 7D).

## 3. Discussion

JNKs represent a signaling hub in many physiological responses and have pivotal functions in cell proliferation, differentiation, development and death [5]. JNKs can be selectively inactivated by dual-specificity phosphatases (DSPs) and transcriptional induction of DSP expression is well documented as a negative-feedback mechanism [40]. In *Drosophila*, this negative feedback loop modulates the level of JNK activity in different developmental processes, such as epithelial sheet expansion (dorsal closure and imaginal disc fusion) [6,17] and morphogenetic death [41].

### 3.1. puc Expression Regulatory Domains

In our analysis of the mechanisms regulating the expression of *puc,* the gene encoding the *Drosophila* JNK-specific DSP [21], we uncovered regulatory sequences (PG2) directing its expression to egg chamber border cells. This expression can also be observed in the *puc* mutant line *puc^B48^* [21] and in diverse protein trap lines [42]. PG2 expands across the first and second introns of *puc*, where the *puc^B48^* insertion is located and its activation depends on JNK activity [25]. All generated PG2 lines show the same expression pattern.

Analyzing in more detail the genomic region spanning *puc* and its surrounding domains, we have found that both in the 5′ and 3′ ends of its transcription unit, regulatory motifs are uncommon. The PG1 and PG5 Gal4 lines just direct reporter expression to salivary glands and posterior spiracles, which most probably constitute unspecific readouts, present in all studied constructs. Their activity in a subset of neurons and a few scattered and unpatterned epidermal cells is minimal. *puc* first and second introns (PG2 line), as previously described [7], activate gene expression in border and follicle cells. Remarkably, they also are involved in very dynamic control of gene expression in distinct glial subpopulations, evolving through embryonic and larval stages. *puc’s* third intron is highly enriched with regulatory domains, affecting, in particular, mesodermal derivatives (PG3), the ring gland (PG4) and different components of the CNS (both domains). Other tissues targeted by the regulatory motifs present in this intron are the epidermis, the tracheal system, the anal pad, the midgut and the maxillary primordia (Table 1).

Although our analysis does not cover every aspect of the newly identified motifs in the *puc* genomic domain, it provides several important clues on the activity and function of the regulatory control mediated by *puc* on JNK signaling: (1) the known expression of *puc* in mesodermal derivatives (body wall muscles and heart), follicle and border cells, and the tracheal system can now be ascribed to identified genomic domains; (2) *puc* expression is modulated by a more complex array of motifs than expected and it targets multiple unforeseen tissues where the role of the JNK pathway has not been previously explored, such as the ring gland, distal tracheal cells, alary muscles or the anal pad; (3) *puc* expression, in many places, appears to be independent of JNK activity, or at least it is not directly targeted by Hep. This has been previously documented for the control of JNK activity by small GTPases in the wing imaginal discs; *hep* is required downstream of Dcdc42 to activate *puc* but the Rac input on *puc* expression is not affected by the absence of *hep* [24]. This suggests that a JNK-related activity is present in discs acting downstream of Rac or that alternative regulatory mechanisms can affect *puc* expression; and (4) eliminating JNK activity (maternal and zygotic *hep* loss of function) can lead to the activation of *puc* expression. We have detected overactivation of *puc* expression in the embryonic epidermis mediated by the RS1 domain in *hep* mutants. Further, in the absence of *hep*, RS2 becomes active in the midgut and in the cells at the edge of the dorsal open embryonic epidermis. RS2 in wild-type animals does not label leading edge epidermal cells (although they are characteristic JNK positive—*puc* expressing cells (*puc^E69^* and *puc^A251.1^* LacZ reporters)). These cells are prompt to die and strongly initiate RS2-dependent expression once JNK signaling is switched off. If this activation could respond to a caspase-dependent but JNK-independent cell death pathway, as the one described in the *Drosophila* eye imaginal discs mediated by Eiger [tumor necrosis factor (TNF)] [43], or to another cascade, remains to be elucidated. RS2 directed expression in the absence of JNK activity is key, as *puc* expression has been employed, on a regular basis, as a readout of JNK-dependent cell death-inducing activity. It might not be correct to assume that the JNK pathway is active every time that *puc* expression is turned on.

The patterns of expression directed by each Gal4 line (PGs and RSs) in embryos and third instar larvae, and on the embryonic and first instar larvae CNS, when relevant, are summarized. Grey boxes relate to the non-specific expression detected in salivary glands and posterior spiracles (although we could not discard them, they may represent bona fide targets). Green boxes highlight expression patterns not observed in the wild-type condition, that are switched on in the absence of JNK activity (*hep^1^* maternal and zygotic condition). Red boxes point to patterns of expression that are eliminated in *hep^1^* mutants.

The JNK pathway does not just play an important role in regulating a wide range of cellular activities, but it is also fundamental for tumor growth [44]. In this scenario, the pathway has been linked to both Ras-induced tumorigenesis and, in association to the Hippo pathway, to tissue growth control [45,46]. The links between these signaling elements are still unclear. In particular, in *Drosophila*, it has been found that JNK signaling can activate or suppress Yorkie depending on context [47]. The identification of these new regulatory elements controlling the expression of *puc* provides a great opportunity to investigate the implication of other regulatory cascades on the negative feedback control of JNK activity in different scenarios.

### 3.2. puc Expression Regulatory Factors

The bioinformatic scan of the RS1 to RS4 regulatory sequences identified a set of transcription factors potentially regulating *puc* expression. Although we cannot comment on all identified factors, we found that some of them show expression patterns reminiscent of those detected in our anatomical descriptions. Others have been functionally linked to processes in which the JNK signaling pathway appears to participate.

Within the RS1 domain, binding sites for members of the Iroquois (Iro) family, such as Araucan (*ara*), Caupolican (*caup*), or Mirror (TALE homeobox transcription factors characterized by an atypical homeodomain) show high relative scores. Their expression patterns, however, seem unrelated to the expression of the RS1 element [48]. Most relevant appears to be the presence of a Dorsal (*dl*) binding site (NF-κB transcription factor with an N-terminal Rel homology domain). Dl acts together with Dif downstream of the Toll pathway, positively regulating hemocyte proliferation. Interestingly, an adjacent Deaf-1 binding site (MYND-type zinc finger factor), showing a very high relative binding score, was also found. Deaf-1 acts downstream of Cactus (IκB homolog) and Dif and Dorsal (NF-κB homologs) regulating the immune response and the expression of Drosomycin, a well-known target of the JNK signaling cascade [49,50,51]. The last factor possibly relevant in this context could be Pangolin (*pan*) (an HMG-domain transcription factor of ubiquitous expression that is a key component of the canonical Wingless signaling pathway). Wingless and JNK signaling have been found to cooperate on dorsal closure, ventral patterning and compensatory cell proliferation amongst other events [52,53].

The RS2 domain displays a very characteristic pattern of expression, largely resembling the PG3 pattern, which includes the healing epithelia, midgut, CNS (neurons and glia), body wall muscles, heart, allary muscles and anal pad (Figure 6). The most relevant binding domain found within RS2 corresponds to a bipartite site shared by the basic leucine-zipper transcription factors Creb (cAMP responsive element binding protein) and Jun [54]. Two Creb genes are encoded in flies; CrebA is expressed in the blastoderm embryo, digestive system and epithelium and in the larval gut, fat body and trachea, while in females is also expressed in follicle cells; meanwhile, CrebB is ubiquitous, although its activity is mostly restricted to the nervous system [55,56]. Jun is expressed in the amnioserosa, embryonic epithelium and motor neurons and in imaginal discs, and it has pleiotropic roles in the CNS (synaptic assembly at neuromuscular junction), eggshell chorion assembly and epithelium morphogenesis (dorsal closure). Jun is a direct target of Bsk (JNK), and it is essential for *puc* expression in different tissues, such as the edges of wounded epidermal tissue and in the dorsal epithelium during dorsal closure [29,57].

*bsk* expression is almost ubiquitous and it regulates cell shape and stress responses amongst other processes. In the context of the RS2, we have shown that the absence of Hep (JNKK) triggers a specific response switching on the RS2 enhancer in epithelial cells at the edge of the open epidermis (Figure 6). It has been shown that Bsk transduces the responses to the TNFα pathway activated by Eiger (*egr*) leading to cell death [58]. It is tempting to speculate that the RS2 regulatory element could be implicated in the response to Egr signaling. Likewise, Bsk exhibits cytoprotective activity in neuronal cells in response to wounding [59] and regulates the activity of Sterol regulatory-element binding proteins (SREBP) in neurons and thereby the accumulation of lipids in glia [60]. It also affects the embryonic expression of *puc* in the VNC and its morphogenesis [61]. The potential function of RS2 on *puc* CNS expression remains to be evaluated.

Besides the Creb/Jun bipartite site, RS2 carries a relevant binding site for Dbx (NK-like homeobox transcription factor), which is expressed in neuroblasts of the VNC, and it is involved in axon guidance and neuron fate commitment. Dbx affects the differentiation of neurons via cross-repressive interactions with the products of Exex and Eve transcription factors [62]. Interestingly, *puc* expression is dynamically regulated in a subset of *eve* expressing neurons during VNC morphogenesis [61] pointing to a hypothetical relationship to RS2 activity.

RS3 specifically drives reporter expression to the tracheal system and the epidermis. This expression, in embryos, is reduced in response to a loss of JNK activity (Figure 7). No regulatory factor with a relative score higher than 90% was found (Table S1). Yet, at lower stringency, binding sites for members of the Iro family and for downstream elements of the *dpp* (TGF) signaling pathway, Mad (MH domain transcription factors containing an N-terminal sequence-specific DNA-binding MH1 and a C-terminal MH2 domain that mediates the formation of oligomeric complexes) and Brinker (a transcriptional repressor), were identified. The *dpp* and JNK signaling pathways have been shown to cooperate in multiple developmental processes [63,64]. In the context of RS3 expression, *ara* and *caup,* on one side, and *mad* and *brinker*, on the other are all expressed in the trachea primordia and the developing tracheal system (*mad* is further expressed on the dorsal trunk branches of the tracheal system), as well as on the embryonic epithelia. All the above factors could certainly be linked to *puc* expression in these tissues mediated by RS3.

Last, RS4 directed activity is limited to a very weak expression at the VNC in embryos and at the maxillary primordia and anal pads and scattered in the tracheal system in larvae. Relevant in this context could be Distal-less (*dll*) (NKL homeobox transcription factor), well known for specifying limb (distal) versus body wall (proximal) fates during embryogenesis, but that it is also expressed in the head epidermis, labial and maxillary sensory complexes [65]. Further, in relation to the observed RS4-directed faint VNC expression in embryos, binding sites for the LIM homeobox transcription factors CG4328 and LIM3 and for the NKL homeobox factor Exex are found adjacent. *CG4328* is expressed in the embryonic brain (ventral midline) and the larval ventral nerve cord and is predicted to be involved in neuron differentiation [66]. *Lim3* is expressed in embryonic/larval neurons and regulates neuronal sub-type identity, including motor neuron identity. *exex* is expressed in and regulates the differentiation of motor neurons that project axons to ventral body wall muscles [67]. Within the CNS, the product of *exex* negatively interacts with the products of *Lim3* and *eve* (see above) to govern neuronal specification and differentiation. *Lim3* and *exex* are expressed in near-identical patterns though neither is required for the activation of expression of the other, although they show a complex epistatic relationship [68]. Mis-expression of *exex* causes a thickening of ventral motor projections (but not dorsal motor projections), defects in longitudinal connectives and collapse of commissures [69]. Mutant embryos have extra eve-expressing neurons in the developing CNS [68].

While this study cannot be considered saturated, does highlight a series of potential regulatory factors that could be fundamental for the understanding of the complex and distinct regulatory networks mediated by the JNK signaling cascade, not just during the development of *Drosophila* but, also, for basic physiological and pathological events common to eukaryotes.

## 4. Methods

### 4.1. Cloning of puc Regulatory Sequences

In order to amplify the upstream, intronic and downstream potential regulatory sequences of *puc* (PGs) we designed five pairs of primers that were employed to amplify fragments of genomic DNA. As the expected fragments are around 4 to 6 Kb long, we used a long template PCR system from Roche. These PCR fragments were first cloned in a PGEM T easy vector. Secondly, we subcloned these fragments in a Gal4 expression vector, the pPTGal [70]. pPTGal is a vector that contains two terminals 5′ L TR and 3′ L TR transposase targets that permit its integration in the fly genome. This vector carries also a *white +* gene marker which will lead to a red eye color on a *white*-background. RSs were directly cloned (after amplification employing appropriate primers) in the Bgl2 site of the pPTGal Vector.

Transgenic flies were generated by injections in *y- w-* embryos with a helper plasmid that contains a transposase gene. The hatched flies were crossed individually with *y- w-* flies. Generally, one female or male potential transgenics with three other *y- w-* flies. To map these insertions in the second or third chromosome, the flies were crossed with a double balancer stock (*w; If/CyO; MKRS/TM6B*).

The coordinates of the *puc* Genomic (PGs) domains and the Regulatory Sequences (RSs) are presented below and referred to the *D. melanogaster* r6.40 Genome database (version FB2021_05) [71]. 3R refers to the right arm of chromosome 3 and the number to the genomic coordinates along the chromosome.

*puc* Genomic 1 (PG1): 3R: 8,099,435–8,105,718; Includes the *puc* promoter and upstream sequences.

*puc* Genomic 2 (PG2): 3R: 8,105,378–8,110,997; Includes the *puc* exons 1, 2 and, partially, 3 and introns 1 and 2.

*puc* Genomic 3 (PG3): 3R: 8,111,181–8,116,293; Includes the *puc* exon 3, partially, and the 5’ half of intron 3.

*puc* Genomic 4 (PG4): 3R: 8,115,938–8,121,127; Includes the *puc* 3’ half of intron 3 and, partially, exon 4.

*puc* Genomic 5 (PG5): 3R: 8,120,573–8,122,620; Includes the *puc* exon 4 and downstream sequences.

Regulatory Sequence 1 (RS1) (94 bp): 3R: 8,106,539–8,106,632

AAAAAATGTAAAGCAAAGCAGGTTTCTCAAGCGGCCTGGCAACGCTGAAAAACCCGCTTTGAAACGACCTTTCGAGTACGAATTCATCGGCACA

Regulatory Sequence 2 (RS2) (127 bp): 3R: 8,112,003–8,112,128

GAGACGCAAAAAAGGGGGCACGAGGTGGGAACGAGGAGAGAACTTTAGCCGTGGAATATAATGCTGACGTCAATCGCATTTTTCCATTTCCATTTTCTATGTCCAAAGCTGTTCATCAAGTATTTTT

Regulatory Sequence 3 (RS3) (58 bp): 3R: 8,112,946–8,113,003

TTGTTGTTGCTGTTGCGTGTTGGCGTTGGCAGACAGCATGGCGGCAGCGGCGTCGTCG

Regulatory Sequence 4 (RS4) (92 bp): 3R: 8,115,816–8,115,911

GGTGACATTTTTGTTGGCCCCCGTGCGCATAATTTCAATATTCGTCAGCCGGCTATTTTTAAAATGCAAAAATCGACCAAGCGAAAAAAAAT

### 4.2. Bioinformatic Analyses

The whole genome comparative analysis to identify conserved regions in or around *puc* in different *Drosophila* species was done using the Vista Point tool (https://pipeline.lbl.gov/cgi-bin/gateway2?bg=dm2&selector=vistapoint, accessed on 1 July 2021) on the *D. melanogaster* Apr. 2004 Genome Release [72].

Potential transcription factors binding to the RS motifs were identified through two procedures *in silico*: first, position-weight matrices of the TRANSFAC database [73] were analyzed on the Match Suite of Genexplain (https://genexplain.com/services/%20match%20tools, accessed on 1 July 2021); second, the JASPAR collection of transcription factor DNA-binding matrices (http://jaspar.genereg.net/, accessed on 1 July 2021) was employed [74]. The JASPAR CORE database is open source and contains a curated, non-redundant set of profiles, derived from published collections of experimentally defined transcription factor binding sites for eukaryotes. For sequence analysis, we utilized the CORE *D. melanogaster* database (153 entries) complemented with a subset of manually selected general eukaryotic transcription factors up to a collection of 229 entries (Table S2). Scanning was performed at an 85% relative profile score threshold.

### 4.3. Drosophila Strains and Genetics

The following stocks were used:

*pJFRC81-10XUAS-IVS-Syn21-GFP-p10* (attP2) (Dr. Todd Laverty, Janelia Research Campus)

*hep^1^; 10XUAS-IVS-Syn21-GFP-p10* (this work)

*w; If/CyO; MKRS/TM6B*

All crosses were performed at room temperature.

### 4.4. Drosophila Embryo Fixation and Dissections

Embryos were collected at 25 °C and aged at 18 °C for 17–19 h, then dechorionated in 100% bleach for 90s. They were washed thoroughly with water and processed. Whole mount embryos were fixed and de-vitellinized according to [75].

*Drosophila* embryo dissections for generating flat preparations were performed according to [76]. Briefly, crosses were maintained in embryo collection baskets at room temperature and synchronized by repetitive changes of juice-agar plates, with a time interval of 2 h. All embryos laid within this period of time were aged for approximately 13 h at 25 °C, or until reaching mid-stage 16 (3-part gut stage). At this point, the chorion was removed with bleach for 1 min and the embryos were rinsed extensively with water. For dissection, dechorionated embryos were transferred with forceps on the surface of a small piece of double-sided tape, adhered on one of the sides of a poly-L-Lysine coated coverslip. After orienting the embryos dorsal side up and posterior end towards the center of the coverslip, the coverslip was flooded with saline (0.075 M Phosphate Buffer, pH 7.2). Embryos were manually de-vitellinized using a pulled glass needle and dragged to the center of the coverslip, where they were attached to the coated glass with their ventral side down. A dorsal incision was performed using the glass needle directed from the anterior to the posterior end of the embryo. The gut was removed by mouth suction and a blowing stream of saline was used to flatten the lateral epidermis. Tissue fixation was done with 3.7% formaldehyde in saline for 10 min at room temperature. After this point standard immunostaining procedures were followed.

### 4.5. Immunohistochemistry

Immunostaining of whole mount or flat-dissected stage 16–17 *Drosophila* embryos was performed using the following primary antibodies: mouse anti-Fas2 (1:100, clone 1D4, DHSB), rabbit anti-GFP tag polyclonal (1:600, Thermo Fisher Scientific), mouse anti-Repo (1:100, clone 8D12 DHSB) and rat anti-Elav (1:1000, clone 7E8A10 DHSB).

The secondary antibodies used for detection were: Goat anti-Rabbit IgG (H + L) Alexa Fluor 488 conjugate (A-11008), Goat anti-Mouse IgG (H + L) Alexa Fluor 555 conjugate (A-21422) and Donkey anti-Rat IgG (H = L) Alexa Fluor 555 conjugate (A-48270). All secondary antibodies were used in a dilution of 1:600 and were from Invitrogen.

### 4.6. Image Acquisition

Immunostained embryos were oriented on Poly-L-Lysine coated slides and mounted in Vectashield anti-fading medium (Vector Laboratories, Burlingame, CA, USA) using a 22 mm × 6 mm glass coverslip (thickness 1) as a spacer between the slide and the overlaying coverslip. Flat-prepped immunostained embryos were mounted in the same way.

Image acquisition was performed on a Zeiss LSM 700 inverted confocal microscope, using a 25× oil immersion lens (1.3 NA) for whole mount embryos and a 40× oil immersion lens (1.3 NA) for flat-dissected embryos respectively. Z-stacks spanning the whole embryo thickness were acquired with a step size of 2 µm.

### 4.7. Larvae Live Imaging

Third instar larvae were anesthetized in a chamber saturated with ether fumes for 8 min at room temperature. Next, they were stretched and aligned with forceps on a piece of double-sided tape, adhered to a glass slide and covered with a 3:1 mix of Halocarbon Oil 27/700 with a Refraction Index of 1.47. Two 22 mm × 6 mm glass coverslip (thickness 1) were used as a spacer between the slide and the coverslip (thickness 1.5). Images were acquired on a Superesolution DRAGONFLY 505 (Andor) installed on an inverted confocal microscope (Nikon Eclipse Ti2) using a 10× multiple immersion lens (0.8 NA). 150 slices with a step size of 2 µm were collected.

## Figures and Tables

**Table 1:** Summary of expression patterns. Shaded in grey are common, unespecific expression patterns. Shaded in green are patterns suppressed in *hep^1^* mutant conditions. Shaded in red are those patterns activated in *hep^1^* mutant conditions.

	Embryo	CNS Embryo/1st Instar Larvae	3rd Instar Larvae
**PG1**	Salivary Glands		Salivary Glands
	Posterior Spiracles		Posterior Spiracles
		Brain lobes Neurons	Denticles precursors
		Thoracic ganglion Neurons	
**PG2**	Posterior Spiracles		Posterior Spiracles
	CNS	CNS Glia	CNS
	Epidermis (patchy)	· Perineural/Subperineural	· VNC subset
	Tracheal System	· Ensheating	· Glia intersegmental nerve
	Foregut		Epidermis
			Tracheal System
			Foregut
			Posterior midgut
			Anal pad
**RS1**	Salivary Glands		
	Posterior Spiracles		Posterior Spiracles
	Hindgut		Epidermis (patchy)
	Maxillary primordia		Tracheal System (patchy)
	Epidermis		Maxillary primordia
**PG3**	Salivary Glands		Salivary Glands
	CNS	CNS Glia	Midgut/Hindgut
	Midgut (weak)	· Repo-negative midline	Body wall muscles
	Body wall muscles	· Surface	Heart
	Pharynx/ring gland	VNC tracheal arborization	Alary muscles
**RS2**	Salivary Glands		Salivary Glands
	Posterior Spiracles		
	CNS	CNS	Body wall muscles (subset)
	Body wall muscles (subset)	· Neurons	Heart
	Healing Epithelia	· Glia	Alary muscles
	Midgut		Anal pad periphery
**RS3**	Salivary Glands		Salivary Glands
	Posterior Spiracles		Posterior Spiracles
	Epidermis		Epidermis
	Tracheal System		Tracheal System
			· Dorsal trunks
			· Distal arborization
**RS4**	Salivary Glands		Salivary Glands
	Posterior Spiracles		Posterior Spiracles
			Tracheal System
			Anal pad
			Maxillary primordia
**PG4**	Salivary Glands		Salivary Glands
	Posterior Spiracles		Posterior Spiracles
	Epidermis (patchy)	Brain lobes/VNC Interneurons	Epidermis (patchy)
	Ring gland	Nerves axonal projections	Ring gland
		Astrocytes	Posterior midgut
		Ensheathing glia	Tracheal junctions
**PG5**	Salivary Glands		Salivary Glands
	Posterior Spiracles		Posterior Spiracles
	Epidermis (scattered)		Epidermis (scattered)
	Maxillary primordia?		

## Data Availability

Not applicable.

## Supplementary Materials

The following are available online at https://www.mdpi.com/article/10.3390/ijms222212205/s1.
