# Supplementary material for: Dissection of the Regulatory Elements of the Complex Expression Pattern of Puckered, a Dual-Specificity JNK Phosphatase
Source: Int J Mol Sci. 2021 Nov 11;22(22):12205. doi: 10.3390/ijms222212205 (PMC8623796; doi:10.3390/ijms222212205)
Supplement: Supplementary file 1 [file ijms-22-12205-s001.zip › Enhancers Paper 6 Sup.pdf]

Dissection of the regulatory elements of the complex expression pattern of Puckered, a dual-specificity JNK phosphatase

Katerina Karkali and Enrique Martin-Blanco*

Instituto de Biología Molecular de Barcelona (IBMB-CSIC)

Parc Cientific de Barcelona, Baldiri Reixac 10-12

Barcelona 08028

Spain

* Corresponding author: embbmc@ibmb.csic.es

SUPPLEMENTARY FIGURES AND TABLES LEGENDS

Figure S1: PG2 CNS expression in larvae

A) General PG2-directed expression in glial cells (perineural/sub-perineural) decorating the overall surface of the thoracic ganglia and ventral segments (arrowhead) of a first instar larva. It is also shown in scattered cells on the surface of the optic lobes (shaded in red). Anterior is up. Scale bar 50 µm. **B**) transversal section across the abdominal segments from the image in **A**. The glial nuclei mostly localize at the ventral surface (arrowhead) but their cytoplasmic extensions cover the intercommissures and the whole surface of the VNC trunk. Dorsal is up. Scale bar 10 µm. **C**) The optic lobe (shaded in red) of a PG2-expressing larvae is shown at high magnification. Glial cells at its surface are highlighted (arrowheads). Anterior is up. Scale bar 10 µm.

Figure S2: RS1-driven expression in embryonic and larval stages

A) Dorsal view of a live stage 17 embryo expressing GFP under the control of the RS1 Gal4 line. Expression is detected in some scattered epidermal cells and in two restricted groups at the anterior and posterior ends of the embryo (arrowheads). Scale bar 100 µm. **B)** Lateral view of a dorsal-open stage 17, *hep* null embryo showing RS1-driven GFP expression in an area around the posterior end and, ectopically, in the epidermis (arrowheads). This expression is not observed in wild type embryos. Scale bar 100 µm. **C** and **D)** Dorsal (**C**) and lateral (**D**) views of a live third instar larva expressing GFP under the control of RS1. The marker is expressed in a group of cells at the anterior end, at the posterior spiracles, scattered dorsal epidermal cells and the dorsal tracheal trunk (arrowheads). Scale bar 400 µm.

Figure S3: PG3 is expressed in midline glia

A and **B**) Flat preparation of embryos at stage 17 expressing GFP under the control of the PG3 Gal4 line. Dorsal (**A**) and ventral (**B**) views of three segments of the ventral nerve cord (VNC) double stained with an anti-Repo antibody labelling most of the glial cells. Arrowhead points to cells expressing GFP belonging to the midline glia. Scale bar 10 µm. Anterior is top. **C** and **D**) Frontal dorsal (**C** - anterior is up) and transverse view (**D** - dorsal is up) of first instar

larvae preparations. Shaded in red are the cell bodies of the dorsal midline glia and in yellow those of the ventral midline. The arrowheads point to the massive expression observed in the tracheal branches penetrating the brain. Scale bar 10 µm.

Figure S4: RS2 is expressed in both glia and neurons

A to **D**) Flat preparation of embryos at stage 17 expressing GFP under the control of the RS2 Gal4 line. Dorsal (A and C) and ventral (B and D) views of three segments of the ventral nerve cord (VNC) are shown. **A** and **B**) Double staining with an anti-ELAV pan-neural antibody. Arrowhead point to individual neural cells co-expressing both markers. **C** and **D**) Double staining with an anti-Repo antibody labelling most of the glial cells. Arrowhead points to cells co-expressing GFP and Repo (Glia). Scale bar 10 µm. In all cases anterior is top. **E** and **F**) Frontal dorsal (**E** - anterior is up) and transverse view (**F** - dorsal is up) of first instar larvae preparations. The arrowheads point to neural cells and the glial extensions colonizing the commissures. Scale bar 10 µm.

Figure S5: RS4-activation pattern

A) Expression of a RS4-directed GFP-reporter on a live late stage 17 embryo. Salivary glands (arrowhead), the posterior spiracles and conspicuous groups of epithelial cells are labelled. Scale bar 100 µm. B) GFP expression as a marker for PG4 activity in whole mount preparations recapitulate that detected live (arrowhead). Scale bar 100 µm. **C** and **D)** Dorsal (**A**) and ventral (**B**) views of a live third instar larva showing RS4-directed GFP expression in the tracheal trunk and the midgut (arrowheads in C), the perimaxillar domain and the anal pad, and weakly at the CNS (arrowheads). Scale bar 400 µm. Anterior is always left.

Figure S6: PG4 expression in neurons in first instar larvae

A) General PG4-directed expression in neurons in a restricted pattern in the brain lobes, the thoracic ganglia and ventral segments (arrowheads) of a first instar larva. Anterior is up. Scale bar 50 µm. **B**) transversal section across the abdominal segments from the image in **A**. The neurons nuclei mostly localize at the dorsal surface (arrowhead) but their axonal extensions develop ventrally but do not cross the midline. Dorsal is up. Scale bar 10 µm. **C**) 3D

reconstruction of the full arborization of the two neurons (arrowhead) expressing PG4 in the optic lobe. Anterior is up. Scale bar 10 µm.

Table S1: Transcription factors (TF) binding to the RS motifs identified by JASPAR

List of TFs putatively recognizing the RS1 to RS4 query sequences (color coded groups) by JASPAR with a Relative score higher than an 85% threshold. Each group was sorted by its internal motif binding position in the query sequencesequence

The JASPAR Matrix ID, Name of the gene, JASPAR internal Score, Relative score (relative frequency between the hit and the matrix motif), Sequence ID (RS1 to RS4 query sequences), Start and End (first and last nucleotide targetted in the query sequences), Strand (in the query sequence recognized by the TF) and Predicted sequence (within the query) are annotated.

Table S2: JASPAR database

The JASPAR database empoyed in the motif scan analysis consist of the CORE *D. melanogaster* database (153 entries) complemented with a subset of manually selected generic eukaryotic transcription factors (TF) up to a collection of 229 entries. The JASPAR ID, name of the gene, species, TF class and family (within these classes) are annotated.
