# Supplementary figures and images for: Dissection of the Regulatory Elements of the Complex Expression Pattern of Puckered, a Dual-Specificity JNK Phosphatase
Source: Int J Mol Sci. 2021 Nov 11;22(22):12205. doi: 10.3390/ijms222212205 (PMC8623796; doi:10.3390/ijms222212205)

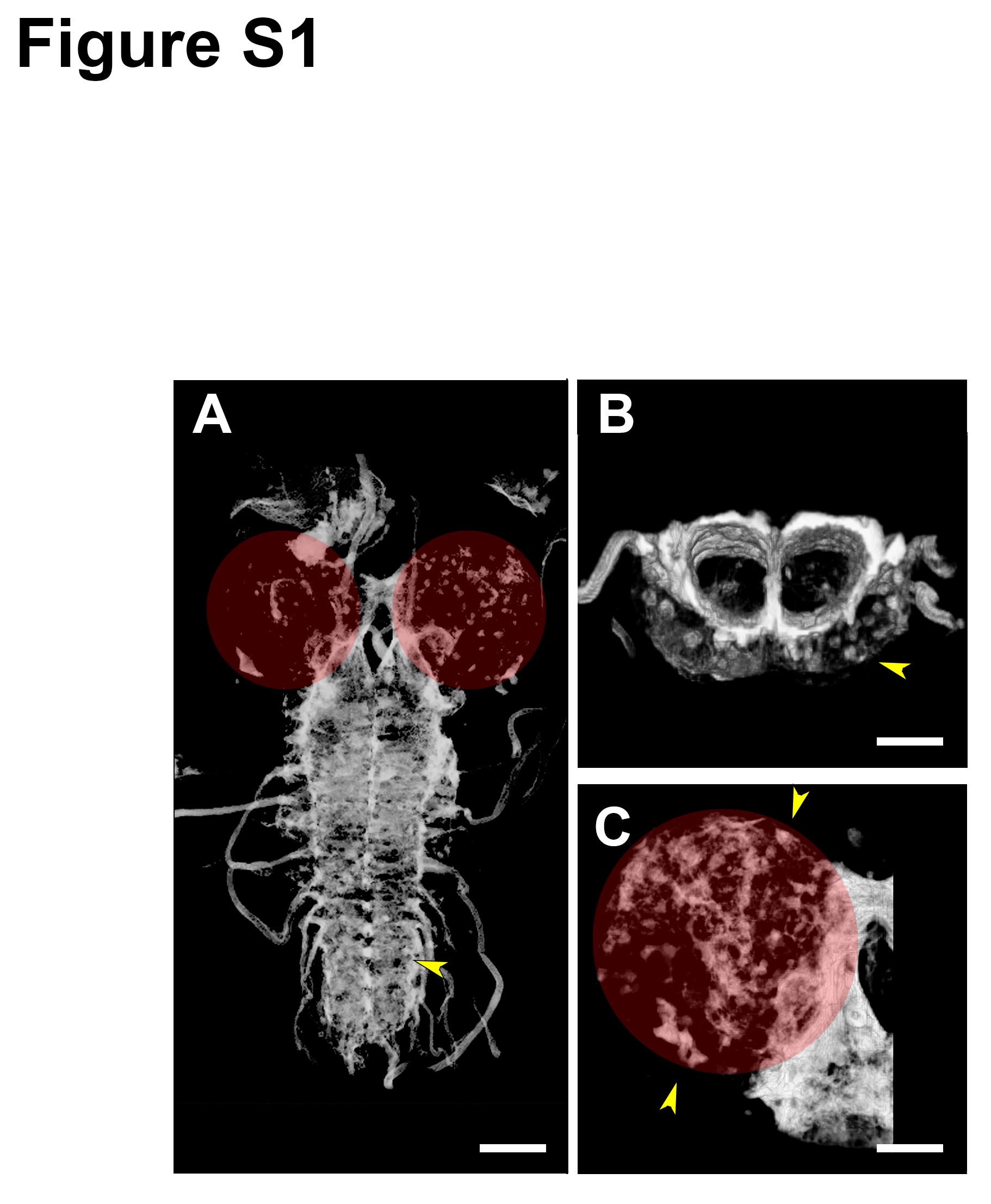

Supplement: Supplementary file 1 [file ijms-22-12205-s001.zip › Figure S1.tif]

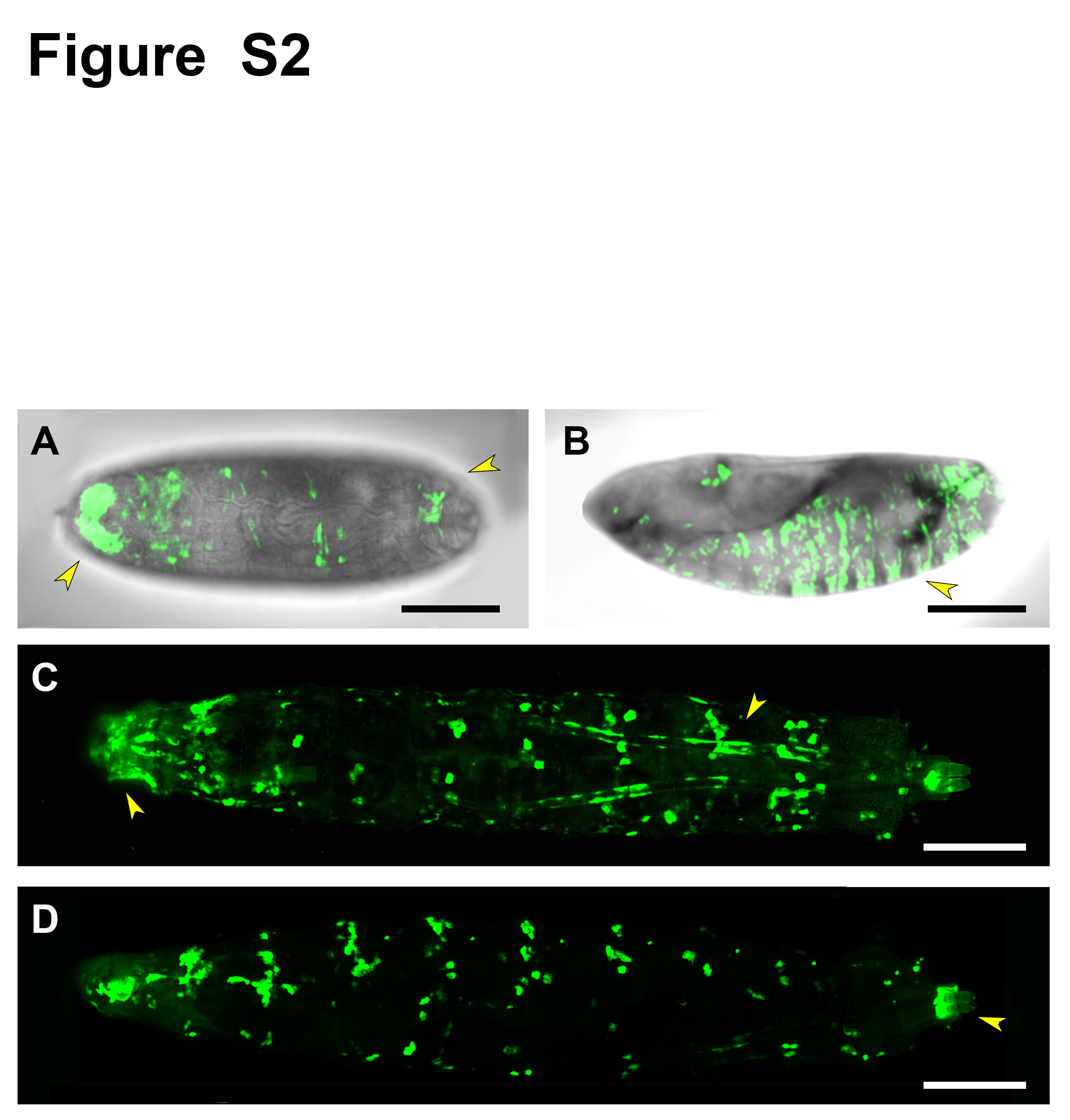

Supplement: Supplementary file 1 [file ijms-22-12205-s001.zip › Figure S2.tif]

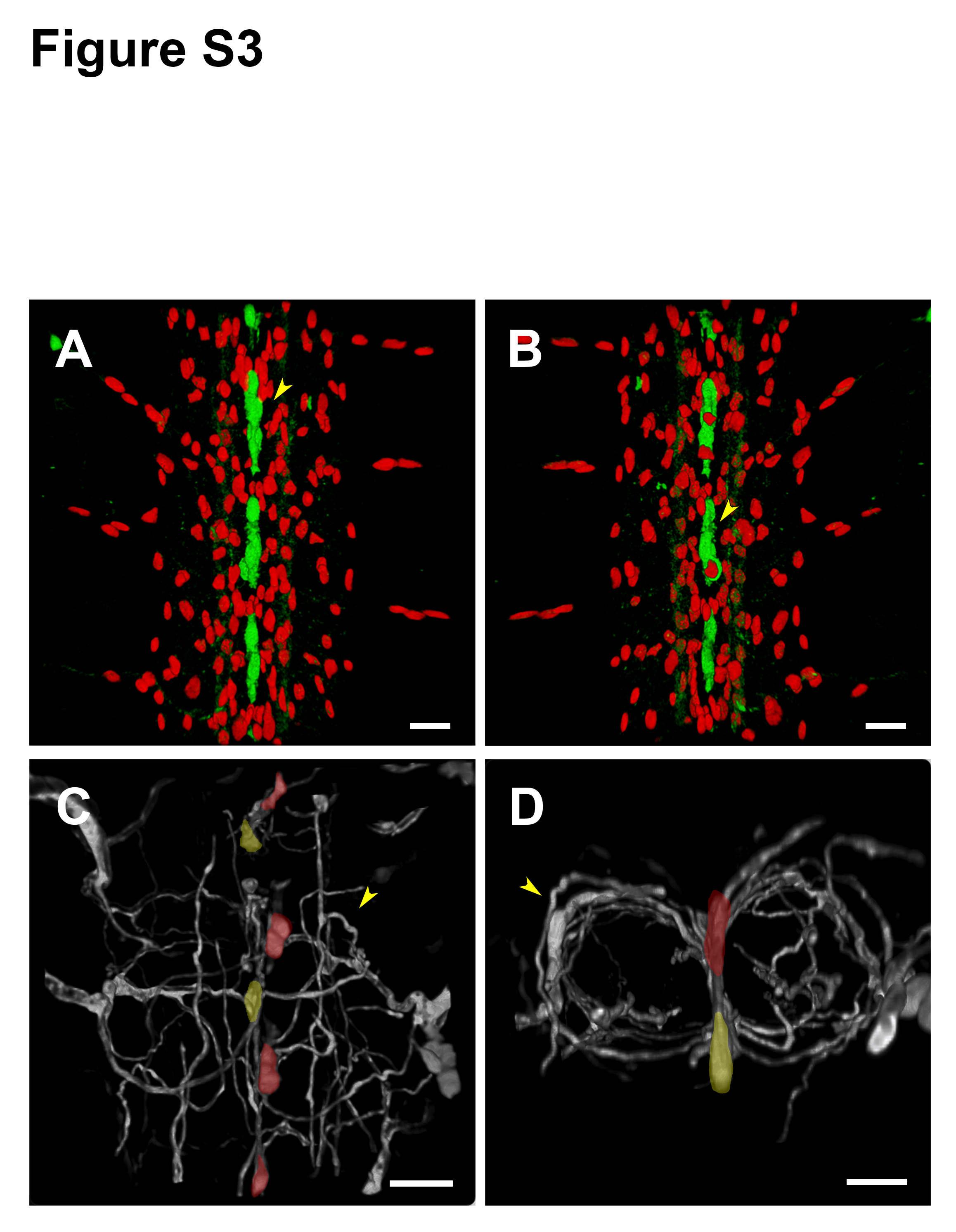

Supplement: Supplementary file 1 [file ijms-22-12205-s001.zip › Figure S3.tif]

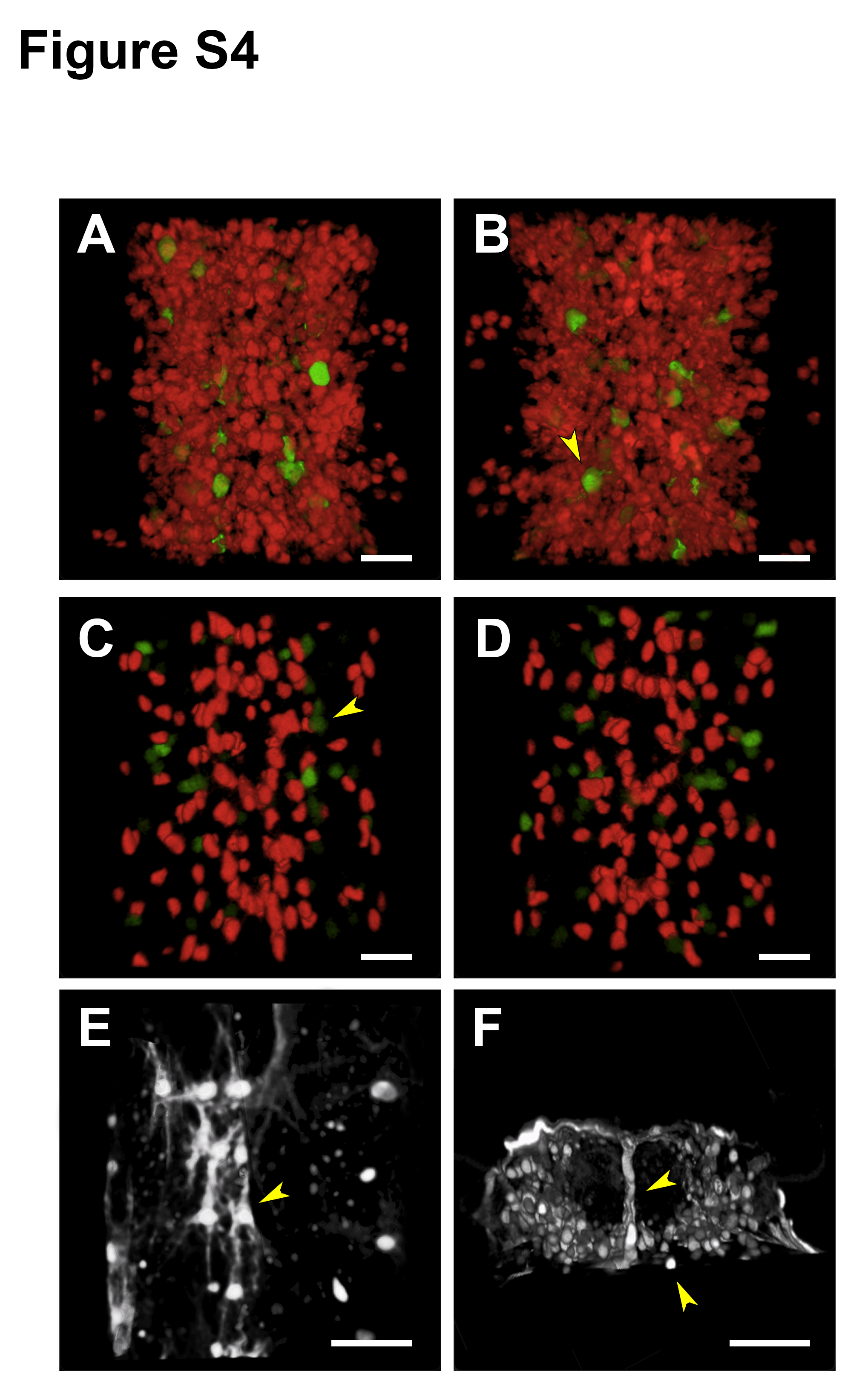

Supplement: Supplementary file 1 [file ijms-22-12205-s001.zip › Figure S4.tif]

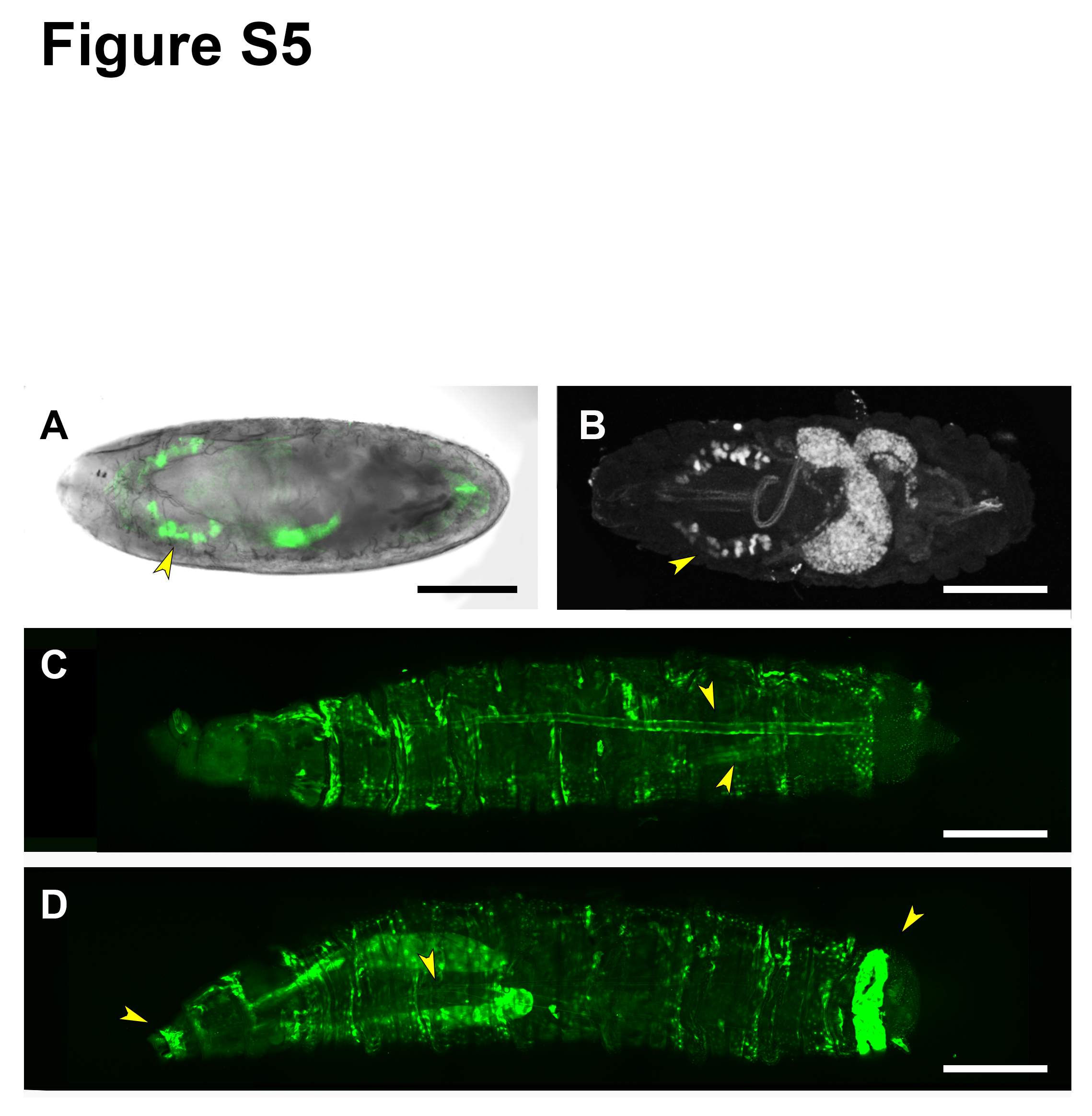

Supplement: Supplementary file 1 [file ijms-22-12205-s001.zip › Figure S5.tif]

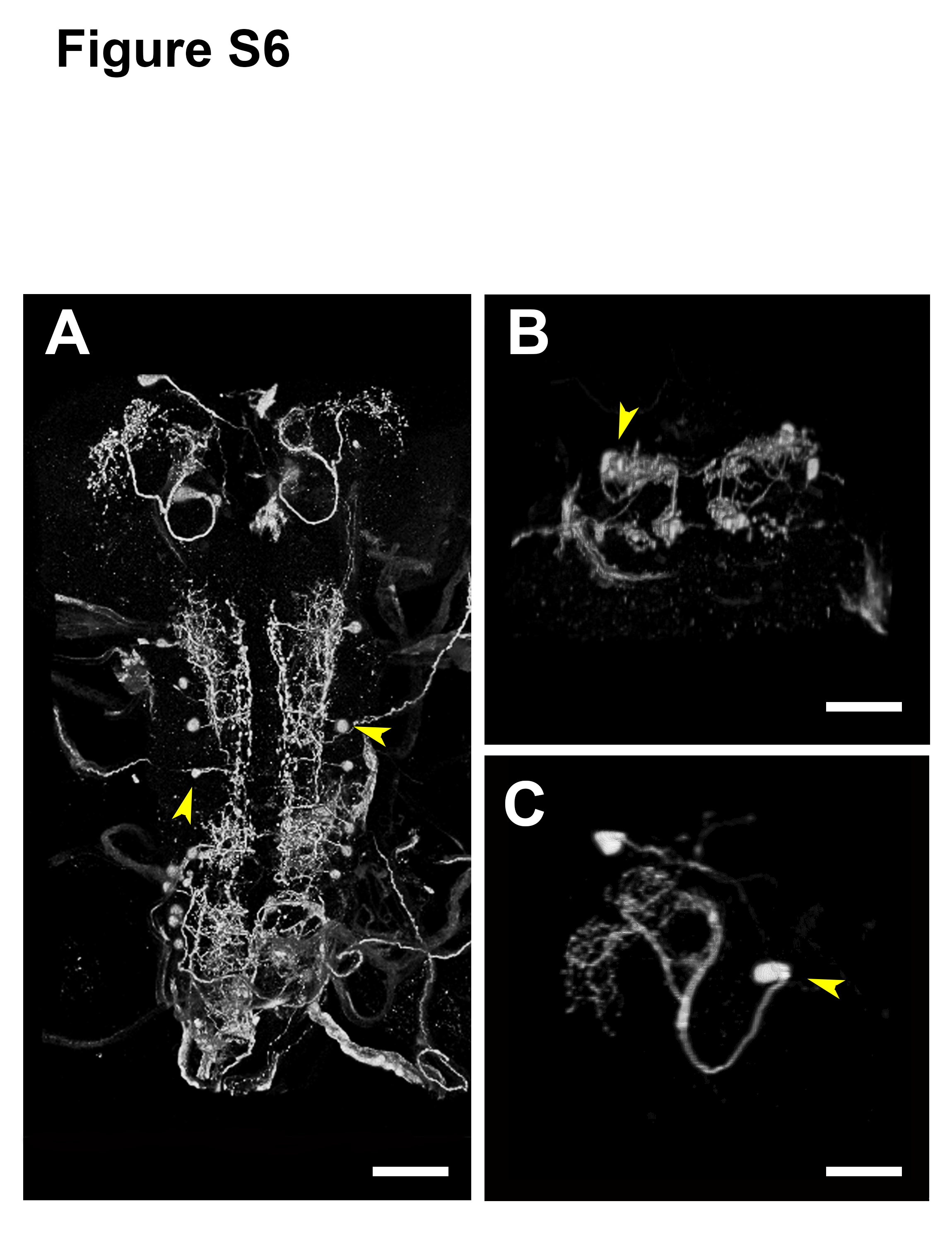

Supplement: Supplementary file 1 [file ijms-22-12205-s001.zip › Figure S6.tif]
